# *Mir-106b* Cluster Regulates Primordial Germ Cells Differentiation from Human Mesenchymal Stem Cells

**DOI:** 10.22074/cellj.2021.6836

**Published:** 2021-07-17

**Authors:** Sadaf Mahboudi, Kazem Parivar, Zohreh Mazaheri, Shiva Irani

**Affiliations:** 1 Department of Biology, Science and Research Branch, Islamic Azad University, Tehran, Iran; 2 Basic Medical Sciences Research Center, Histogenotech Company, Tehran, Iran

**Keywords:** Mesenchymal Stem Cells, MicroRNA, *MiR-106b*

## Abstract

**Objective:**

Numerous evidence indicates that microRNAs (miRNAs) are critical regulators in the spermatogenesis process. The aim of this study was to investigate *Mir-106b* cluster regulates primordial germ cells (PGCs) differentiation from human mesenchymal stem cells (MSCs).

**Materials and Methods:**

In this experimental study, samples containing male adipose (n: 9 samples- age: 25-40 years) were obtained from cosmetic surgeries performed for the liposuction in Imam Khomeini Hospital. The differentiation of MSCs into PGCs was accomplished by transfection of a lentivector expressing *miR-106b*. The transfection of *miR- 106b* was also confirmed by the detection of a clear green fluorescent protein (GFP) signal in MSCs. MSCs were treated with bone morphogenic factor 4 (BMP4) protein, as a putative inducer of PGCs differentiation, to induce the differentiation of MSCs into PGCs (positive control). After 4 days of transfection, the expression of *miR-106b*, STELLA, and *FRAGILIS* genes was evaluated by real-time polymerase chain reaction (PCR). Also, the levels of thymocyte differentiation antigen 1 (Thy1) protein was assessed by the western blot analysis. The cell surface expression of CD90 was also determined by immunocytochemistry method. The cytotoxicity of *miR-106b* was examined in MSCs after 24, 48, and 72 hours using the MTT assay.

**Results:**

MSCs treated with BMP4 or transfected by *miR-106b* were successfully differentiated into PGCs. The results of this study also showed that the expression of miR-106b was significantly increased after 48 hours from transfection. Also, we showed *STELLA, FARGILIS*, as well as the protein expression of Thy1, was significantly higher in MSCs transfected by lentivector expressing *miR-106b* in comparison with MSCs treated with BMP4 (P≤0.05). MTT assay showed *miR-106b* was no toxic during 72 hours in 1 µg/ml dose, that this amount could elevated germ cells marker significantly higher than other experimental groups (P≤0.05).

**Conclusion:**

According to this findings, it appears that *miR-106b* plays an essential role in the differentiation of MSCs into PGCs.

## Introduction

Infertility is a serious physiological problem in human populations, especially in young adults. Epidemiological studies have showed that, male infertility accounts for approximately 50% of all causes of infertility among couples (1). Transplantation of stem cells for infertility has attracted many attention of researchers in recent years. Germ cells are differentiated cells that contribute to the complicated processes of fertilization. To date, many researchers have devoted themselves to reproducing germ cell differentiation, or gametogenesis, *in vitro* (2). It has been established that mesenchymal stem cells (MSCs) which are mainly derived from bone marrow or adipose tissues have great potentials (3) for the repair of various types of tissues. MSCs can differentiate into bone, neurons, adipose, cartilage, muscle, hepatocytes, insulin-producing cells, and skin in proper conditions* in vivo* (3-5). Also it is stated that MSCs have been regarded as an attractive and promising tool for cell-based therapy in immune disorders and inflammatory diseases, as well as for regenerative medicine, owing to their potent immunomodulatory function, paracrine effects and capacity of multilineage differentiation. Previously, other researcher show that generation of spermatogonial stem cells (SSCs) from MSCs *in vitro* (6).

Furthermore, stem cells can be readily isolated, they have high proliferation rates and high potentials for the differentiation into various types of cells. Based on these features, they could be valuable to be applied for autologous transplantation. Nayernia et al. (7) demonstrated that murine bone marrow stromal cells (BMSCs) are able to differentiate into early germ cells *in vitro* and *in vivo*. Also, Cakici et al. (8) recently demonstrated that adipose tissue-derived mesenchymal stem cells (ASCs) which were probed by green fluorescent protein (GFP) are capable differentiating into sperm-like cells that could lead to the recovery of fertility in a rat model of busulfan-treated azoospermia (9).

It has been shown that BMP4 and retinoic acid are frequently employed for the differentiation of MSC into spermatogonial cells. However, only a small proportion of cells would be able to differentiate, or in the case of differentiation, they would not be capable of continuing the spermatogenesis process. Recently researchers have been focused on short sequences of micro RNAs for the differentiation of MSCs into different lineages of cells.

MicroRNAs can regulate the expression of the vast majority of proteins at post-translational level by miRNA-induced silencing complex (miRISC). This complex is able to bind their target mRNAs, and then it degrades the synthesized mRNAs, leading to the silencing of a particular gene. The silencing of genes is an essential biological phenomenon by which numerous cellular processes including self-renewal, proliferation, differentiation, and apoptosis could be fine-tuned (10). Moreover, studies have reported that miRNAs are highly expressed and they are involved in the process of spermatogenesis (11-17). In line with this study, the loss of DICER (a protein which facilitates the activation of the RISC activation) could be resulted in a defect in germ cell development (18, 19). Tong et al. (20), characterized the active miRNAs involved in the development of spermatogonial cells by the microarray method. These researchers identified the profile of a number of miRNAs in undifferentiated spermatogonial cells (THY1+-enriched). 

Other study showed that Mirlet7 family plays a significant role in the spermatogonial differentiation (19). Also other reports indicated that both *miR-17-92* (miRc1) cluster and its paralog *miR-16b-25* (miRc2) cluster contribute to the self-renewal of SSCs and the promotion of the proliferation of undifferentiated spermatogonial cells. The spermatogonial differentiation depends on several intrinsic and extrinsic signaling proteins, modulate the expression of the leading genes. The downregulation of *LIN28, MYC, MYCN, miR-17-92* (*miRc1*), and *miR-106b-25* (*miRc3*) promotes the differentiation of the undifferentiated spermatogonial cells (16). The field of biotechnology has a tremendous and pivotal contribution to the manipulation of cellular contents to obtain the desired outcomes in biological events. The transfection of cells with miRNAs is one of the exemplary strategies for the overexpression/downregulation of a particular miRNA to alter cellular behaviors. This strategy has become an important tool in miRNA-based therapeutics (21). The goal of the current research has been focused on the role of the *miR-106b* cluster in the differentiation of adipose-derived MSCs (ADMSCs) into PGCs independent of the use of BMP4. The corresponding miRNA was overexpressed in ADMSCs for 4 days to induce the differentiation of these types of cells.

## Materials and Methods

### Ethics statement

In this experimental study, the perusala case-control was approved by the Human Ethics Committees of Azad University (Code number: IR.IAU.SRB.REC.1396.71). The adipose tissue were removed and transferred under the approved protocols to the research laboratory. All efforts were made under sterile conditions.

### Cell isolation and culture

Samples containing male adipose (n: 9 samples- age: 25-40 years) under local anesthesia were obtained from cosmetic surgeries performed for the liposuction in Imam Khomeini Hospital (all subjects signed an informed consent). Samples were washed several times in phosphate buffered saline (PBS, Gibco, Germany). Then, the tissues were minced and treated with an equal volume of 0.075% type I collagenase (Sigma, Germany) with continuous agitation at 37˚C for 1 hour. The enzyme activity was neutralized with Dulbecco’s Modified Eagle Medium (DMEM) high glucose without glycerophosphate (Sigma, Belgium) solution containing 10% fetal bovine serum (FBS, Gibco, UK) and then centrifuged at 1200 ×g for 10 minutes to obtain a high-density cell pellet (Clinical Benchtop Centrifuges). The resultant supernatant was discharged, and stromal vascular fraction (SVF) pellet was mixed with 2,000 µl DMEM solution using a pipette. The suspended cells were subsequently passed through 100 µm nylon filter mesh (Falcon Company, USA) and incubated at 37˚C in 5% CO_2_ in DMEM solution containing 10% FBS. The medium was replaced every 2 days.

### Characterization of adipose-derived stem cells by Flow cytometry

ADSCs were washed three times in PBS and then centrifuged (Hettich, Germany) at 400 g for 5 minutes and resuspended in ice cold PBS. For the blockade of nonspecific bindings, the cells were rinsed with 10% bovine serum albumin (BSA, Gibco, UK) in PBS for 30 minutes, washed three times in PBS, and incubated with mouse anti-human CD90 (Abcam, Germany), Rabbit anti-human CD105 (Abcam, Germany), Rabbit anti-human CD34 (Abcam, Germany) and rabbit anti-human CD45 (Abcam, Germany), Mouse anti-human CD44, Mouse anti-human CD73 as a primary antibody at 4˚C for 1 hour. Then, the primary antibodies were washed three times in PBS at room temperature and incubated with goat anti-rabbit IgG conjugated with FITC and goat anti-mouse IgG conjugated with phycoerythrin (PE) as a secondary antibody at a ratio of 1:100 at 37˚C for 30 minutes in the dark. Afterward, the cells were washed twice in PBS, centrifuged at 400 g for 5 minutes, and evaluated by flow cytometry (Olympus, Japan). The percentage of positive cells was calculated with respect to the negative control. The isotype antibody was applied in negative controls.

### Osteogenic differentiation

To induce the differentiation of ADSCs (at the fourth passage) into osteogenic cells, the culture medium of ADSCs changed to osteogenic maintenance medium containing 10 mM β-glycerophosphate, 0.2 mM ascorbic acid, and 7-10 M dexamethasone for 21 days (all chemicals were purchased from Sigma, UK). Cells in a culture medium were nourished every three days throughout the study. To confirm the differentiation of osteogenic cells, Alizarin Red S stain was used. Briefly, the osteogenic medium was removed and washed three times in PBS. The cells were fixed in 70% ethanol at 4˚C for 1 hour. After the fixation process, cells were rinsed in deionized water and air-dried. The fixed cells were stained with 2% Alizarin Red S (pH=7.2, Sigma, Belgium) at 37˚C for 1 hour, washed in deionized water, and photographed under an inverted microscope (Olympus, Japan).

### Adipogenic differentiation

ADSCs at the fourth passage were incubated for 21 days with adipogenic maintenance medium containing 50 μg/ml indomethacin, 50 μg/ml ascorbic acid, and 100 nM dexamethasone (all chemicals were purchased from Sigma, Germany). The medium changed every three days. The adipogenic differentiation was confirmed using Oil Red O (Sigma, Germany) staining. Briefly, the adipogenic medium was removed and washed three times in PBS. The cells were fixed in 10% formalin for 30-60 minutes at room temperature, washed in distilled water, and treated with 2 mL isopropanol (60%) for 5 minutes. Then, they were removed and stained with Oil Red O (2 mL to each well) at room temperature for 5 minutes. Finally, the cells were rinsed in tap water and photographed under an inverted microscope (Olympus, Japan). 

### Study design

The induction of PGCs differentiation was performed based on previously research (22). At the fourth passage, the sub-confluent culture of MSCs maintained in DMEM solution supplemented with 10% FBS. Forty-eight hours prior to the induction of PGCs differentiation, media were replaced with pre-induction media consisting of DMEM, 20% FBS, and 25 ng/ml BMP4 (BME; Sigma, St. Louis, MO, Germany). To induce the PGCs differentiation and enrichment, the pre-induction media were removed, and the cells were washed with PBS. After that, cells were transfected by a lentivector expressing *miR-106b*. The percentage of PGCs-like cells was calculated in 10 randomly chosen fields under an inverted microscope. Each experiment was carried out triplicate.

### MiR-106b transfection

A lentiviral vector expressing *miR-106b* was procured from Gene Copoeia Inc. The lentivirus containing *miR-106b* and its control vector was purchased from Biosettia (USA). The lentivirus was generated regarding the User Manual of theLenti‐Pac™ HIV Expression Packaging Kit (GeneCopoeia, Inc.). For the transfection of ADSCs with lentivirus, 1×10^6^ ADSCs were seeded on the plates, and 20 µl of virus suspension (MOI of 50) was added to the plates. The *miR-106b* and its negative control were transfected into pre-inducted ADSCs using lipofectamine 3000 (Invitrogen, USA), in accordance with the manufacturer’s instructions. The cells were transferred to a plate, and cultured in 5% CO_2_ at 37˚C for 4 days.

### Reporter gene assay

Hek293 cells were infected with lentivirus carrying the *miR-106b* for 2 days. The GFP activity was monitored 24 hours after the transfection using the fluorescent microscopy assay system (Labo Med, USA). The GFP activity was considered as an internal control.

### Cell cytotoxicity assay

To determine the cytotoxicity of *miR-106b* transfection in MSCs, the cell viability was measured using MTT (3-(4, 5-Dimethylthiazol-2-yl)-2, 5-diphenyltetrazolium bromide (Atocel, Austria). Briefly, 1× cells were seeded on 96 well-plates and incubated at 37˚C overnight to allow the cells to adhere. The cells were then treated with multiple concentrations of *miR-106b* including 0, 0.25, 0.5, and 1 µg of the corresponding miRNA. After the incubation, cells were incubated with MTT solution (5 mg/ml) for 4 hours at 37˚C and then the medium was removed to solubilize formazan crystals. Afterward, 100 µl dimethyl sulfoxide (DMSO, Merck, Germany) was added to each well, and the absorbance was measured using an ELISA reader (Bio-Rad Laboratories, USA) at an excitation wavelength of 570 nm. The percentage of viability was evaluated by the comparison of the absorbance of treated cells with the control cells. 

### Immunocytochemistry

Cultured PGCs were fixed with 4% paraformaldehyde, incubated with primary antibody, at a dilution of 1:100, against CD90 (Santa Cruz Biotechnology, Santa Cruz, CA, USA) at 4˚C overnight. Then, the cells incubated with secondary antibody conjugated with FITC at room temperature for 1 hour. DAPI (Sigma, Germany) was applied for the staining of the cell nucleus. The antibody against CD90 was used at a 1∶100 dilution.

### Western blot analysis

Cells were harvested and lysed in lysis buffer (RIPA, Beyotime Institute of Biotechnology, China) supplemented with protease inhibitors (PMSF, Aladdin). The equal amounts of protein (40 μg) were separated by sodium dodecyl sulfate polyacrylamide gel electrophoresis (SDS-PAGE) with 5-12% Tris-Glycine gel (Invitrogen, USA) and subjected to standard western blot analysis. Antibodies against THY1 (Santa Cruz, USA) and β-actin (Santa Cruz, USA) were diluted at 1∶1,000. Secondary antibodies used for the western blot analysis were goat anti-mouse IgG-HRP (Santa Cruz, USA) or goat anti-rabbit IgG-HRP (Santa Cruz, USA). Enhanced chemiluminescence was performed according to the manufacturer’s instructions (Amersham Life Sciences Inc., Arlington Heights, IL). The results were subjected to densitometry analysis using the ImageJ software. To ensure equal amounts of protein were loaded, the β-actin protein was employed as an internal control. The relative protein expression level was defined as the ratio of the expression of the target proteins to the GAPDH expression level.

### MiRNA target genes prediction by quantitative real-time polymerase chain reaction analysis 

Total RNA, including miRNAs, was extracted using the mirVana miRNA Isolation kit (Ambion, USA) according to the manufacturer’s instructions. The *miR-106b* was detected using RT^2^ miRNA First Strand Kit (SA Biosciences, USA). The specific miRNA and U6 primers purchased from QIAGEN were used for real-time polymerase chain reaction (PCR). The relative expression was determined using the comparative Ct method (2^-ΔΔCt^). The expression of mRNAs was determined using SYBR green real-time PCR assay. The levels of mRNA expression were normalized to that of the *GAPDH* expression as the loading control. The relative expression was calculated using the comparative Ct method (2^-ΔΔCt^). Table 1 shows the primers used for real-time PCR. 

**Table 1:** The sequences of primers used for evaluation of relative


Gene	Primer sequencing (5ˊ-3ˊ)	Accession number

*FRAGILIS*	F: CATGTCGTCTGGTCCCTGT	NM_003641.4
	R: GTGGAGGCATAGGCCTGG	
*GAPDH*	F: TTCAGCTCTGGGATGACCTT	NM_002046.7
	R: TGCCACTCAGAAGACTGTGG	
*STELLA*	F: GGTTCGGAATAAGGCAAAGAG	NM_182489.1
	R: AGGTGAGATACCAAGGGGAGG	


### Statistical analysis

The statistical analysis of the obtained data in quantitative parts was performed using the SPSS software version 16 (SPSS, Chicago, IL). The independent sample t test method or One Way ANOVA were applied for the comparison of the results between groups. The level of significance was set at P<0.05. The data was represented by mean ± SD. All of data was repeated 3 times. Qualitative data of the cell culture and differentiated part of experiment was described in the text as same as immunostaining and western blot result.

## Results

### Adipose-derived stem cell culture

**Fig.1:**
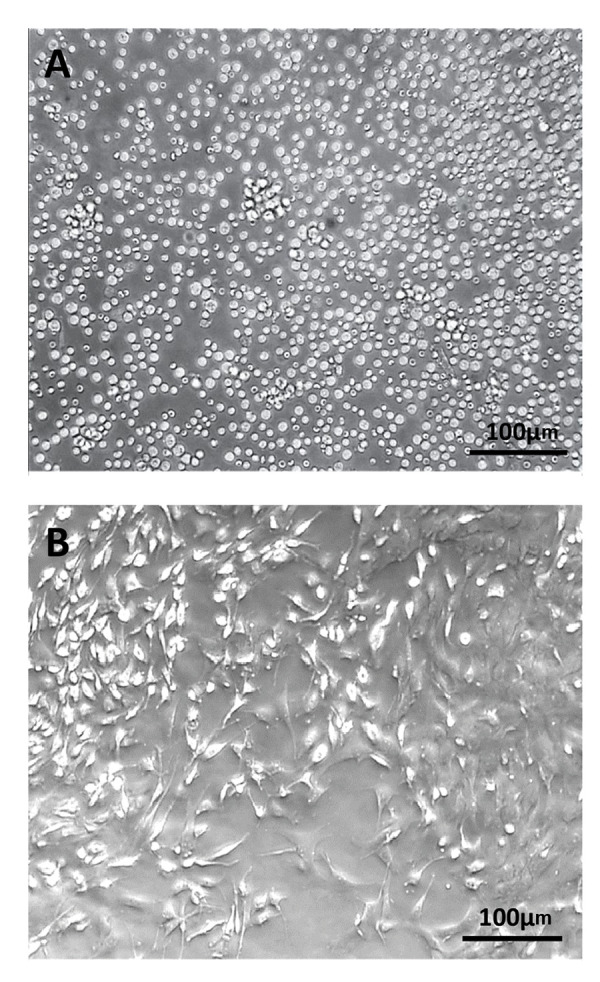
The cells isolated from ADSCs. **A.** Isolated stem cells 4 hours after incubation and **B.** ADSCs in the 4^th^ passage (scale bar: 100 µm). ADSCs; Adipose-derived stem cells.

ADSCs adhered to plastic flask similar to bone marrow MSCs which were characterized by a rapid proliferation. At earlier hours, the cells were floating, and their nucleus was visible (Fig.1A). After 24 hours, the floated cells were adhered to dish to form fibroblast-like colonies. ADSCs formed spindle-like shape (fibroblast-like) and were loaded with several lipid granules within those cells. The lipid granules attached to each other and created large droplets; then, released into the cells culture medium. The first was made in 7 days when the cells reached confluence. After the first passage, the cells showed extensive proliferative capacity passage. The four passages were performed in 13 days, and then, the cells were used for the differentiation experiments (Fig.1B).

### Adipose-derived stem cell characterization and differentiation

**Fig.2:**
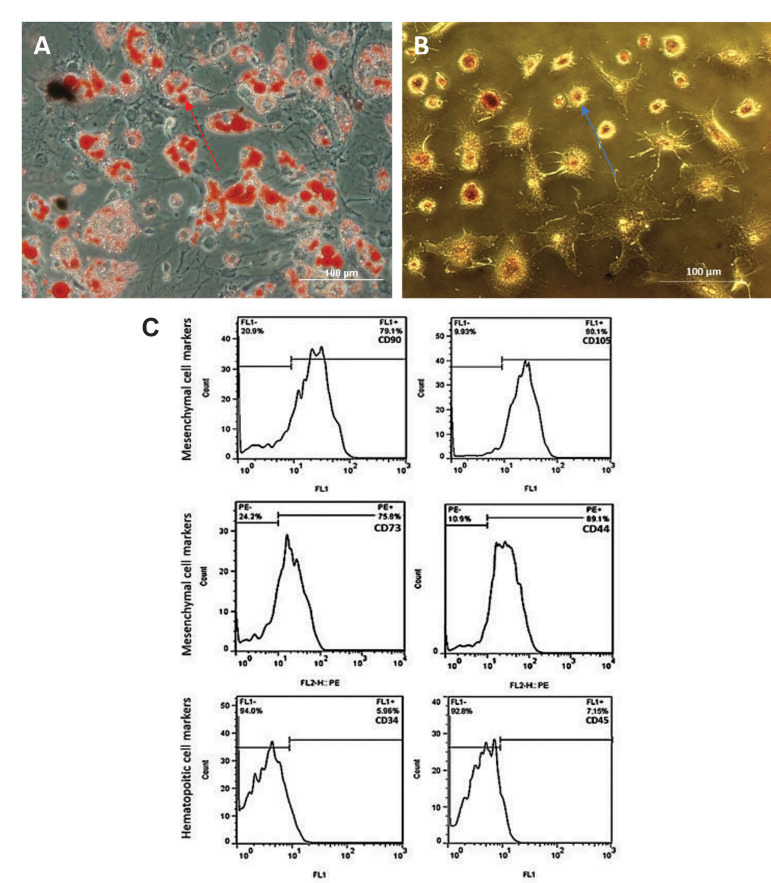
The* in vitro* osteogenesis and adipogenic differentiation. **A. **Adipose-derived stem cells (ADSCs) after incubation for 21 days in the adipogenic differentiation medium. The cells were visualized with Oil Red O staining, **B. **ADSCs after incubation for 21 days in the osteogenic differentiation medium. The cells were visualized with Alizarin Red S stain. The blue arrow indicates osteoblasts, and the red arrow shows adipocytes and the accumulated fat droplets (scale bar: 100 μm). **C.** Cell surface markers: CD90=79.1 ± 5.73, CD105=90.1 ± 3, CD73=75.8 ± 3.61, CD44=89.1 ± 6.49, CD34=5.98 ± 1.64, and CD45=7.15 ± 0.26. The number of positive cells for each marker was assayed by flow cytometry. The data was presented as mean ± SD.

As illustrated in Figure 2A, B, ADSCs showed the differentiation potential into adipogenic and osteogenic linages while they were induced by adipogenic and osteogenic maintenance media, respectively. The adipogenic differentiated cells were visualized with Oil Red O stain. The red arrow in Figure 2A shows adipocytes and the accumulated fat droplets. The osteogenic differentiated cells were visualized with Alizarin Red S stain. The blue arrow in Figure 2B indicates osteoblasts. Furthermore, ADSCs were characterized by their cell surface antigens. As shown in Figure 2C, a high percentage of the studied cell population were expressing the specific markers of mesenchymal stem cells including CD90 (79.1 ± 5.73), CD105 (90.1 ± 3), CD73 (75.8 ± 3.61), and CD44 (89.1 ± 6.49). The expression of the specific markers for hematopoitic stem cells was detected by few cells (CD34=5.98 ± 1.64, and CD45=7.15 ± 0.26).

### Cell cytotoxicity assessment

**Fig.3:**
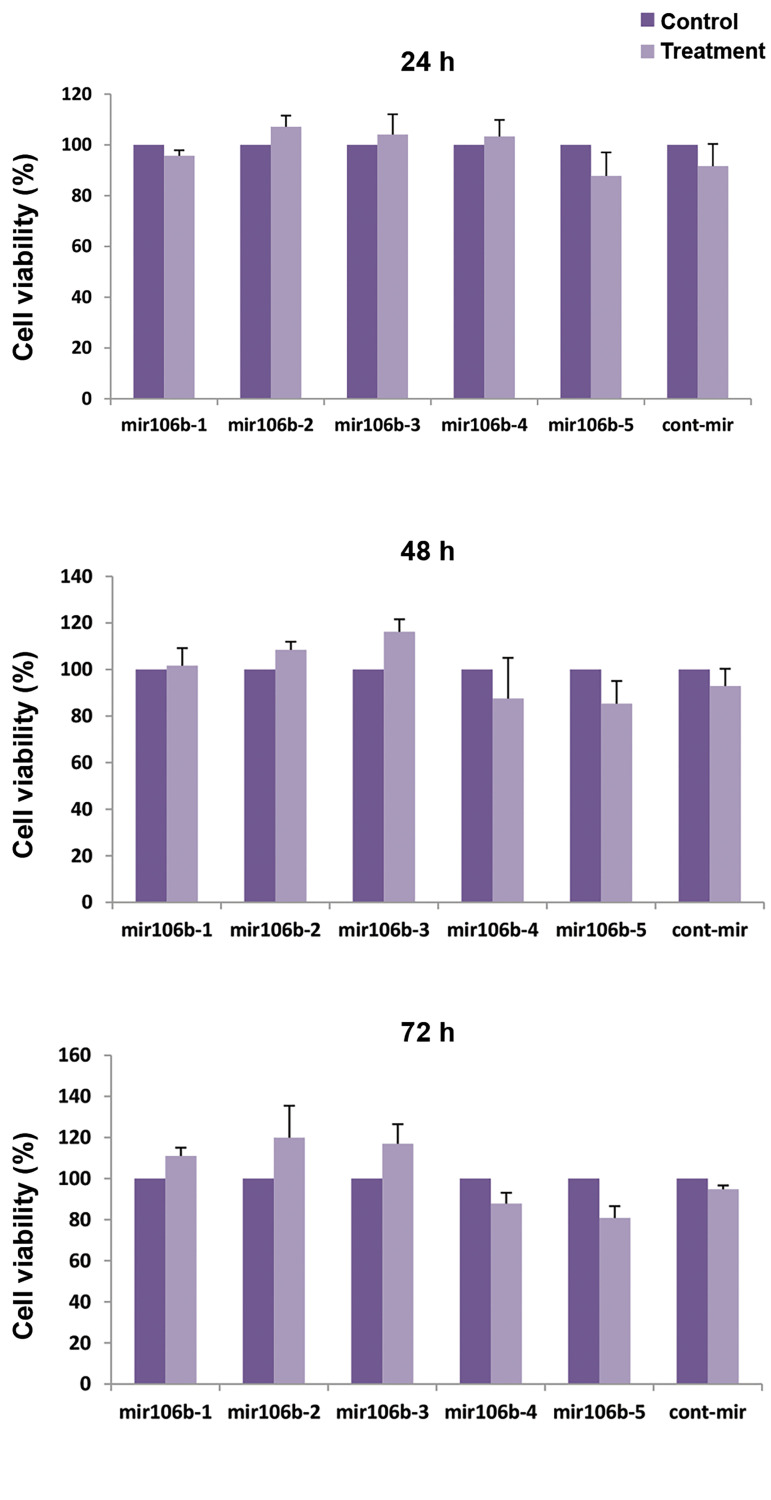
Cytotoxicity of *miR-106b* expressing MSCs. The cytotoxicity level of *miR-106b* expressing MSCs was evaluated after 24, 48, 72 hours incubation at various concentrations of *miR-106b*. MSCs; Mesenchymal stem cells and h; Hour.

In order to investigate thecytotoxicity of *miR-106b* transfection, MTT assay was conducted to examine the viability of ADSCs. According to Figure 3, after 24, 48, and 72 hours incubation time, no significant reduction was observed in the viability of cells expressing *miR-106b* compared with the control cells lacking *miR-106b*. 

### Primordial germ cells induction from mesenchymal stem cells

**Fig.4:**
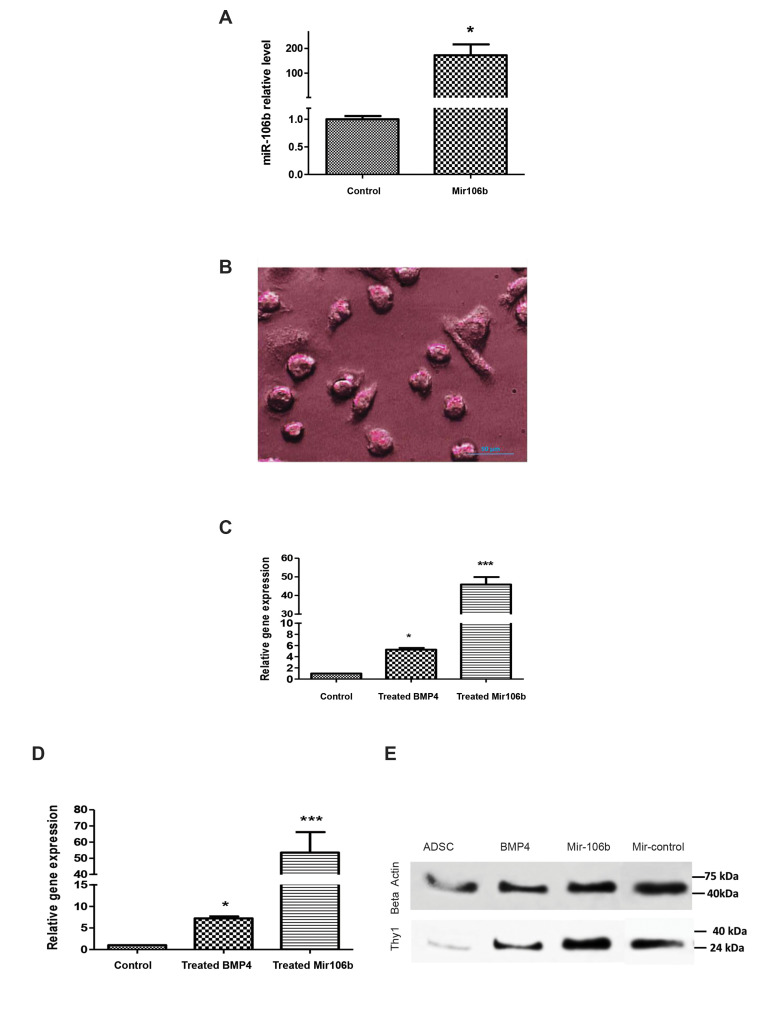
The differentiation of ADSCs into PGCs. **A.** The expression of the *miR-106b* measured by real-time PCR after the transfected by lentivector expressing *miR-106b*. **B. **Alkaline phosphatase-positive cells (scale bar: 50 µm). **C, D. **The expression of *STELLA* and *FRAGILIS* genes and E.Thy1 protein levels were evaluated as specific differentiation markers using real-time PCR and western blot analysis, respectively. ADSCs; Adipose-derived stem cells, PGCs; Primordial germ cells and PCR; Polymerase chain reaction. * demonstrates the significant changes in comparison to control (*; P≤0.05 and ***; P≤0.0001).

**Fig.5:**
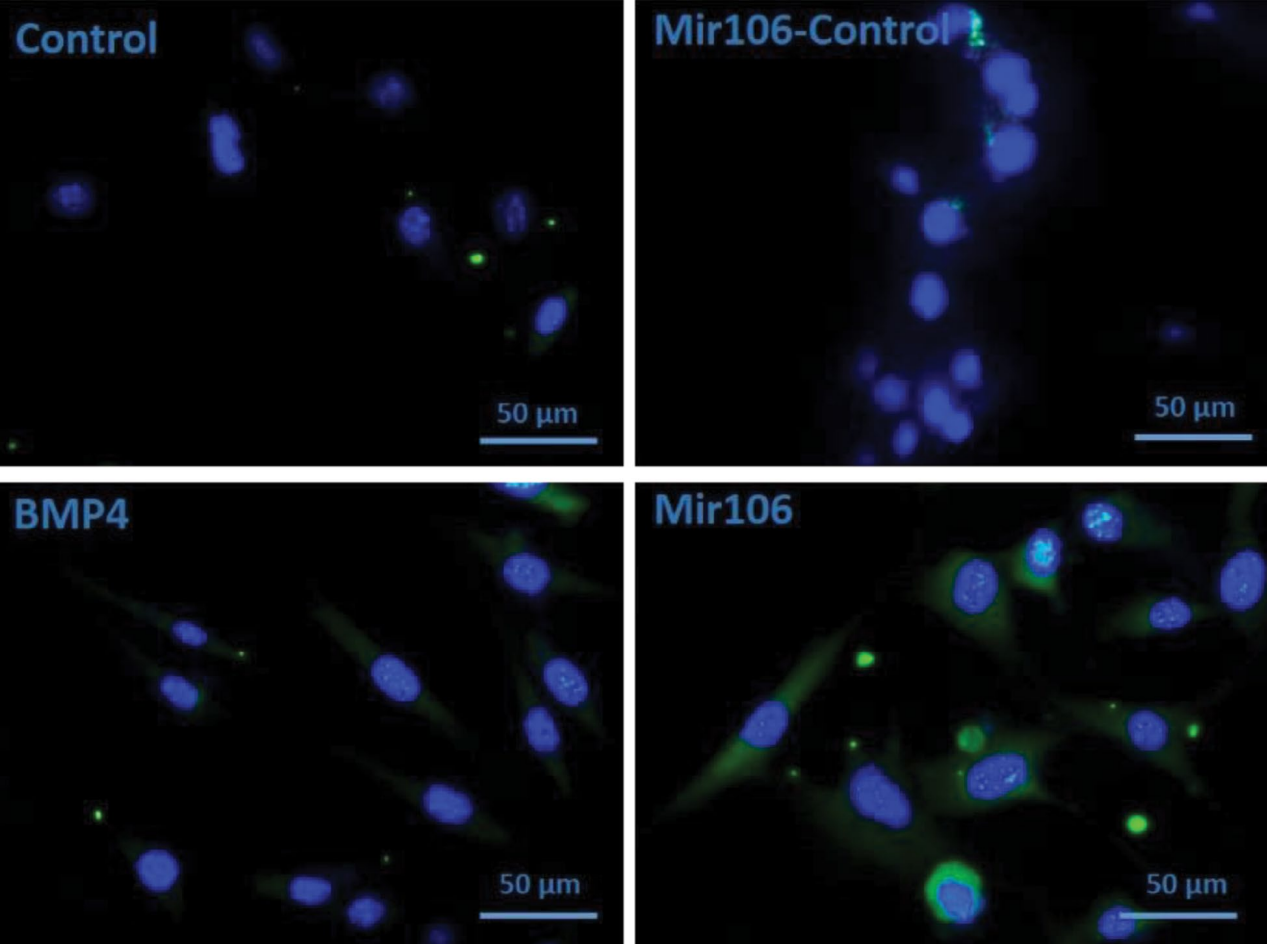
CD90 protein level measured as a marker of differentiation on germ cells (ADSCs) (scale bar: 50 µm).

The results showed that three of four specific differentiation markers (FRAGILIS, Thy1, and STELLA) were significantly increased at the levels of gene and protein when the *miR-106b* was overexpressed (Fig.4A). Alkaline phosphatase expression was showed in cells transfected by the *miR-106b* (Fig.4B). Photograph showed positive alkaline phosphatase staining of differentiated cells. A smaller alkaline phosphatase negative cell, possibly a contaminating undifferentiated MSCs. Figure 4C and D indicate the expression levels of *STELLA* and *FRAGLIS* genes were significantly unregulated in BMP4-, and *miR-106b*-treated cells compared to control cells. Moreover as illustrated in Figure 4E, the amount of THY1 protein was significantly increased in *miR-106b*-treated cells compared to BMP4-treated cells and contr Furthermore, as shown in Figure 5, the expression level of CD90 protein was significantly higher in cells transfected with the *miR-106b* than the cells treated with BMP4. CD90 expression was expressed around the stained nucleus by DAPI (4′,^6^-diamidino-2-phenylindole) in differentiated cell surface. Based on the cells that were stained and non-stained around the nucleus, the results showed over expression of CD90 marker in immunostaining assessment.

## Discussion

The potential capacity of somatic stem cells to differentiate into PGCs, SSCs (23, 24), or advanced spermatids through the meiosis process under appropriate culture conditions has been well-established in the literature (25, 26). The clinical value of direct differentiation might be more valuable than other strategies as the gene transfection creates an imbalance in the gene contents of genetically modified cells during spermatogenesis. However, the molecular mechanisms underlying the germ lineage differentiation from MSCs remain elusive.

The miRNAs are known to regulate the development of germ cells (27). To understand the regulatory role of miRNAs in the development of PGCs, ADMSCs were differentiated into PGCs in which *miR-106b* was transfected into ADMSCs to facilitate the differentiation of these cells into PGCs through the upregulation of some target genes responsible for the development of the cell differentiation. Hence, an *in vitro* model of *miR-106b*-transfected ADSCs was employed to induce the differentiation of these cells into PGCs thereby influencing the proliferation, morphogenesis and protein localization of the corresponding cells. Our findings indicate that the transfection of MSCs with *miR-106b* can by itself increase the specific markers of PGCs namely *STELLA* and *FRAGILIS* genes, as well as the expression of Thy1 protein when compared with MSCs treated with BMP4. Moreover, the surface expression of CD90 was higher in cells transfected with miR-16b than the cells treated with BMP4. Numerous reports have indicated that miRNAs are potentially able to induce the differentiation of MSCs into various tissues. In line with this, an *in vitro* study performed by Sluijter et al. (28) indicated that a number of miRNAs are involved in the proliferation and differentiation of cardiomyocyte progenitor cells (CMPCs). They showed that that *miR-1* and *miR-499* can regulate the proliferation of human CMPCs, as well as their differentiation into cardiomyocytes. Previous studies have also highlighted that miRNAs play critical roles in the process of neurogenesis. Jiao et al. (29) reported that miR-124 promotes the neural differentiation of the subventricular zone, which is the most substantial neurogenic niche in the brain of adult mammalian species. Also, it has been shown that miR-23b induces the chondrogenic differentiation of human MSCs through the suppression of protein kinase A (PKA) signaling (30).

The overall importance of miRNA signaling for the regulation of spermatogenesis has been further elucidated using a conditional knockout of *Dicer gene* in germ cells. The silencing of the *Dicer1 gene* in pro-spermatogonia at the early-stage of the birth using *Ddx4* promoter-driven Cre expression resulted in altered meiotic progression increased apoptosis in pachytene spermatocytes, reduced number of round-shape spermatids, and morphological defects in spermatozoa (31). In a study performed by Holt et al. (32), they revealed that nine newly identified miRNAs including miR-10b, -18a, -93, -106b, -126-3p, -127, -181a, -181b, and -301, which are all exclusively expressed in PGCs according to their comparative study, profiling the miRNA expression of PGCs at 12.5 days post-coitum (dpc), gonocytes (GCs) at 15.5 dpc, SSCs at 5 days post-partum (dpp), and testes at four weeks. BMP4 signaling acts through the Smad family proteins and requires a ligand-specific co-receptor TGF-b (transforming growth factor-Beta) in murine SSCs (33). In agreement with an indirect mechanism, Okamura et al. (34) have shown that the deficiency of PGCs in embryos knockout for BMP4 can be compensated by the activation of a sub-type of type I BMP receptor named Activin A Receptor type 1 (ACVR1) in the visceral endoderm, but not the epiblast where PGC precursors are present. It has been implicated that the expression of *FRAGILIS* is elevated in the migratory PGC, stimulating the expression of other germ cell-specific genes such as *VASA* and *STELLA* (35). *STELLA* is considered a crucial marker for murine PGCs, while* DAZL* and *DDX4 *begin their expression in murine PGCs from around the E10.5 stage and last to be expressed afterward (36). It has been reported that *miR-106b* can activate the Wnt/beta-catenin signaling pathway as the loss of *WNT5A* disrupts murine PGCs migration and male sexual development in mice (37).

## Conclusion

*In vitro* model of the spermatogenesis development are noticed by many researchers. This study developed a new approached to gain PGCs from MSCs by the transfection of ADMSCs with the *miR-106b* lentivector. Upregulatin of *miR-106b* caused to the specific gene markers of the PGC expression, more efficient than the conventional method used by BMP4. It is thought that finding of pathways governing the meiotic and post meiotic cells would shed light on our understanding about the essential molecules involved in the spermatogenesis and its progression.
